# Development and validation of prognostic models for anal cancer outcomes using distributed learning: protocol for the international multi-centre atomCAT2 study

**DOI:** 10.1186/s41512-022-00128-8

**Published:** 2022-08-04

**Authors:** Stelios Theophanous, Per-Ivar Lønne, Ananya Choudhury, Maaike Berbee, Andre Dekker, Kristopher Dennis, Alice Dewdney, Maria Antonietta Gambacorta, Alexandra Gilbert, Marianne Grønlie Guren, Lois Holloway, Rashmi Jadon, Rohit Kochhar, Ahmed Allam Mohamed, Rebecca Muirhead, Oriol Parés, Lukasz Raszewski, Rajarshi Roy, Andrew Scarsbrook, David Sebag-Montefiore, Emiliano Spezi, Karen-Lise Garm Spindler, Baukelien van Triest, Vassilios Vassiliou, Eirik Malinen, Leonard Wee, Ane L. Appelt, on behalf of the atomCAT consortium, Richard Adams, Muhammad Amin, Nikola Dino Capocchiano, Peter Colley, Andrea Damiani, Viola De Luca, Charlotte Deijen, Antri Demetriou, Michael J Eble, Matthew Field, Loukia Georgiou, Ann Henry, Joanna Lau, Mark Lee, John Lilley, Patricia Lopes, Christina Maria Lutz, Stefania Manfrida, Jenny Marsden, Carlotta Masciocchi, Joseph Mercer, Lars Nyvang, Elisavet Papageorgiou, Gareth Price, Thomas Rackley, Mariachiara Savino, Joep Stroom, Ioannis Stylianou, Nilesh Tambe, David Thwaites, Maciej Trojanowski, Vincenzo Valentini, Sandra Vieira

**Affiliations:** 1 Leeds Institute of Medical Research at St James’s, University of Leeds, Leeds, UK; 2 Department of Medical Physics, Oslo University Hospital, Oslo, Norway; 3 MAASTRO (Dept of Radiotherapy), GROW School of Oncology and Developmental Biology, Maastricht University and Maastricht University Medical Centre+, P. Debyelaan 25, 6229 Maastricht, Netherlands; 4 The Ottawa Hospital and the University of Ottawa, Ottawa, Canada; 5 Weston Park Hospital, Sheffield, UK; 6 Fondazione Policlinico Universitario A. Gemelli IRCCS, Università Cattolica S.Cuore, Rome, Italy; 7 Department of Oncology, Oslo University Hospital, and Institute of Clinical Medicine, University of Oslo, Oslo, Norway; 8 Ingham Research Institute and Liverpool Hospital, Liverpool, New South Wales Australia; 9 Addenbrooke’s Hospital, Cambridge, UK; 10 The Christie NHS Foundation Trust, Manchester, UK; 11 RWTH Aachen University Medical Centre, Aachen, Germany; 12 Oxford University Hospitals NHS Foundation Trust, Oxford, UK; 13 Champalimaud Foundation, Lisbon, Portugal; 14 Greater Poland Cancer Centre, Poznań, Poland; 15 Hull University Teaching Hospitals NHS Trust, Hull, UK; 16 Leeds Teaching Hospitals NHS Trust, Leeds, UK; 17 Cardiff University, Cardiff, UK; 18 Aarhus University Hospital, Aarhus, Denmark; 19 The Netherlands Cancer Institute-Antoni van Leeuwenhoek (NKI-AVL), Amsterdam, The Netherlands; 20 Bank of Cyprus Oncology Centre, Nicosia, Cyprus

**Keywords:** Anal cancer, Squamous cell carcinoma, Chemoradiotherapy, Distributed learning, Federated learning, outcome modelling, Overall survival, Locoregional control, Freedom from distant metastasis

## Abstract

**Background:**

Anal cancer is a rare cancer with rising incidence. Despite the relatively good outcomes conferred by state-of-the-art chemoradiotherapy, further improving disease control and reducing toxicity has proven challenging. Developing and validating prognostic models using routinely collected data may provide new insights for treatment development and selection. However, due to the rarity of the cancer, it can be difficult to obtain sufficient data, especially from single centres, to develop and validate robust models. Moreover, multi-centre model development is hampered by ethical barriers and data protection regulations that often limit accessibility to patient data. Distributed (or federated) learning allows models to be developed using data from multiple centres without any individual-level patient data leaving the originating centre, therefore preserving patient data privacy. This work builds on the proof-of-concept three-centre atomCAT1 study and describes the protocol for the multi-centre atomCAT2 study, which aims to develop and validate robust prognostic models for three clinically important outcomes in anal cancer following chemoradiotherapy.

**Methods:**

This is a retrospective multi-centre cohort study, investigating overall survival, locoregional control and freedom from distant metastasis after primary chemoradiotherapy for anal squamous cell carcinoma. Patient data will be extracted and organised at each participating radiotherapy centre (*n* = 18). Candidate prognostic factors have been identified through literature review and expert opinion. Summary statistics will be calculated and exchanged between centres prior to modelling. The primary analysis will involve developing and validating Cox proportional hazards models across centres for each outcome through distributed learning. Outcomes at specific timepoints of interest and factor effect estimates will be reported, allowing for outcome prediction for future patients.

**Discussion:**

The atomCAT2 study will analyse one of the largest available cross-institutional cohorts of patients with anal cancer treated with chemoradiotherapy. The analysis aims to provide information on current international clinical practice outcomes and may aid the personalisation and design of future anal cancer clinical trials through contributing to a better understanding of patient risk stratification.

**Supplementary Information:**

The online version contains supplementary material available at 10.1186/s41512-022-00128-8.

## Background

Anal cancer is a rare disease encompassing only approximately 0.3% of all cancer cases across the world [1, 2], but with a gradually increasing incidence [3]. A combination of radiotherapy and chemotherapy has been established as the standard treatment for localised disease for the last three decades [4–6]. This treatment confers relatively good outcomes, with 75% overall survival rates reported at 5 years [7–10]. Despite this, it has proven challenging to determine the optimal therapeutic radiotherapy dose and to further improve disease control [11–13].

A study by Shakir et al. [14], which analysed data from 385 patients with anal cancer treated in five UK centres with conformal radiotherapy techniques, reported that the site of primary disease was the most common site of relapse (83.4% of cases). In addition, the majority of patients experienced locoregional failure prior to getting metastatic disease. This emphasises the need to establish an effective treatment for locoregional control with an optimal radiotherapy dose. Even though ongoing prospective clinical trials [13] are focusing on this issue, clinical data acquired through standard practice can also be analysed for the development and validation of prognostic models, to further inform clinical practice [15, 16].

Prognostic and predictive models have been proposed in cancer research for more than 20 years [17] and have a wide range of potential applications, including prediction of cancer susceptibility [18, 19], recurrence risk [20, 21] and survival [22–24]. In particular, prognostic models can be used as decision support tools in the clinic, assisting clinicians in making informed decisions about patient management following a diagnosis [25]. However, developing robust prognostic models for anal cancer is particularly challenging. Due to the rarity of the cancer, it can be difficult to obtain sufficient data for robust model training and validation. In addition, ethical barriers and data protection regulations often limit the ability to share data between centres and thus render multi-centre model development unfeasible [26]. A novel data analysis methodology called distributed learning (DL) [27] has paved the way towards model development between institutions and across international borders.

Distributed learning, also sometimes referred to as federated learning, is a privacy-preserving approach that facilitates the development of robust statistical models using data distributed over multiple sites [28]. The main premise of this approach is that no individual-level patient data leaves the originating centre; only non-identifiable aggregated statistics (model coefficients and fit errors) are exchanged between institutions and a central server. Consequently, adopting this methodology minimises privacy issues related to patient data sharing since it does not breach data privacy barriers. DL algorithms operate in an iterative process where the local dataset in each centre is used to calculate local model coefficients and fit errors. These are sent to the central server, where a single globally-convergent model is determined by minimising the total error [29]. This methodology is applicable for the development of models with a relatively small number of patients [27], but has also been proven to be upscalable to more than 20,000 patients [30].

A DL approach may be ideally suited for prognostic modelling in rare cancers such as anal cancer. It could facilitate acquisition of sufficient patient data from multiple international centres with the aim of developing robust generalisable models, while working around many of the barriers associated with physical data sharing. The feasibility of this approach in anal cancer has previously been demonstrated in the atomCAT1 proof-of-concept study [31]. Using data from three international radiotherapy centres, a Cox proportional hazards model for overall survival was trained and validated in a distributed fashion. The study analysed one of the largest available cohorts of patients with anal cancer treated with conformal radiotherapy and carried out robust multi-centre validation of outcome predictors. However, the analysis was limited to a single outcome only (overall survival), whereas other clinically important outcomes such as locoregional control and freedom from distant metastasis were not taken into consideration.

The atomCAT2 (Anal cancer Treatment Outcome Modelling with Computer Aided Theragnostics) study aims to develop prediction models for anal cancer outcomes after chemoradiotherapy through distributed learning. To achieve this, a consortium of 18 international cancer treatment centres based in the UK, Europe, Australia and Canada has been formed. A cohort of more than 1000 patients will be analysed to develop and validate models for overall survival, locoregional control and distant metastasis, as well as to identify key prognostic factors and their effect size. This will provide unique insights and may aid the personalisation of treatment according to each patient’s unique characteristics.

## Methods

### Study design and patient population

This is a retrospective multi-centre cohort study, investigating outcomes after primary (chemo) radiotherapy for anal squamous cell carcinoma (ASCC). The inclusion and exclusion criteria are summarised in Table 1. Patient data will be extracted and organised within the informatics infrastructure at 18 participating radiotherapy centres, where subjects have consented to treatment with chemoradiotherapy. Routine and standard of care data will be used, and no prospective data collection will be explicitly carried out for the purpose of this study. Using a pragmatic approach, centres will be encouraged to include data for all patients treated in their centre fulfilling the inclusion criteria (Table 1). However, pre-existing patient cohorts, representing a subset of available patient cases, will be accepted. Future expansion to more participating centres internationally is planned.

**Table 1:** Participant inclusion and exclusion criteria

**Inclusion criteria**	
•Radical intent external beam radiotherapy treatment for primary anal squamous cell carcinoma, with or without concomitant chemotherapy	
•Radiotherapy delivered using modern radiotherapy techniques (3D-CRT, IMRT or VMAT)	
**Exclusion criteria**	
•Palliative treatment	
•Prior pelvic radiotherapy	
•Brachytherapy (either primary or as boost treatment)	

*3D-CRT* Three-dimensional conformal radiation therapy, *IMRT* Intensity-modulated radiation therapy, *VMAT* Volumetric modulated arc therapy

Patients have been treated according to each participating centre’s protocol, which may include radiotherapy only or varying chemoradiotherapy regimens. Centres will be asked to briefly outline their main treatment and follow-up protocols as part of study participation.

### Outcome definitions

Three outcomes will be explored: overall survival, locoregional control and freedom from distant metastasis. These were identified as key outcome research measures in anal cancer by the CORMAC initiative [32].

### Overall survival

Overall survival will be calculated in days from the first fraction of radiotherapy to either event or censoring, whichever happens first. An event is defined as death from any cause at any point during follow-up. Patients will be censored at the last clinical follow-up date if alive.

### Locoregional control

Time to locoregional control will be calculated in days from the first fraction of radiotherapy to either event or censoring, whichever happens first. An event is defined as any of the following as a first event: (1) Abdominoperineal resection to control locoregional disease at any point during follow-up. This will always take precedence in terms of date for locoregional recurrence. (2) Locoregional disease progression, during treatment or in follow-up (irrespective of whether complete or partial response have been initially achieved), not managed by surgery. This will preferably be confirmed with biopsy, in which case the date of biopsy will count, but will alternatively be based on imaging and clinical examination only (date of imaging will be used). (3) Lack of complete response (non-clearance of disease) at 26 weeks (6 months) from first fraction of radiotherapy, as defined by clinical examination, imaging and/or biopsy [33]. In case of uncertainty or where limited information is available, the date where treatment failure or locoregional recurrence is first noted in the patient records will be used.

Patients will be censored at death, at last clinical follow-up, if undergoing abdominoperineal resection for non-disease related reasons (e.g. due to treatment complications), or in case of distant metastases.

The site of failure (primary tumour versus pelvic/initially involved nodes) will be noted to allow for separate analysis of local and locoregional failure. Failures in pelvic lymph nodes (inguinal, perirectal, internal iliac or external iliac nodes) or in lymph nodes which were part of the original treatment volumes (which may be the case, e.g. for common iliac or para-aortic lymph nodes) will be defined as locoregional failures.

### Freedom from distant metastasis

Freedom from distant metastasis will be calculated in days from the start of radiotherapy to either event or censoring, whichever happens first. An event is defined as distant disease recurrence (previously untreated lymph node metastasis outside the pelvis, or other metastatic sites such as lung, liver, bone) as a first event. This may be confirmed with biopsy, in which case the date of biopsy will count as the date of recurrence, or alternatively based on imaging (date of imaging will be used). In case of any uncertainty or where limited information is available, the date where distant progression is first noted in the patient records will be used. Site(s) of failure will be noted. Patients will be censored at local recurrence, at death, or at last clinical follow-up.

### Identification of relevant prognostic factors

Already-established prognostic factors for the outcomes in question have been identified through a systematic review of the literature [34]. Studies published after 2000 which reported on disease-related outcomes and examined prognostic factors in multivariable analysis for overall survival, locoregional control, and freedom from distant metastasis were reviewed. In these studies, at least 70% of patients were treated with conformal radiotherapy techniques (3D-defined targets on computed tomography (CT), beams conformed to targets, e.g. using multi-leaf collimators (MLCs), 3D dose calculation and optimisation of dose distributions). This approach identified the initial list of relevant data to be collected; this was subsequently reviewed by three senior clinical oncologists with expertise in anal cancer treatment, and additional factors were added.

### Data collection and completeness

Relevant patient data will be identified and extracted from existing research and clinical databases. Data extraction from databases will be carried out in an automated fashion where possible, with additional manual review if needed. Each participating centre will be responsible for ensuring good data quality by spot checking all extracted data to identify any outliers and to make sure the coding system used is correct, according to the data dictionary provided. Data items are classified as either “essential” or “optional”. For “essential” data items, centres will aim for at most 10% missing data for any given data item across their study cohort. If more than 10% of data is missing for an individual data item, imputation techniques will be implemented according to the framework set out below (see “Missing Data” section). For “optional” data items, missing data will be accepted. Each centre will contribute data from a minimum of 40 patients to ensure a representative sample and achieve a reasonable balance of patient heterogeneity, as well as limit reporting of subgroups with one or only a few patients. See Additional file 1 for full definition and coding of data items.

### Missing data

For outcome data, complete case analysis will be used for each of the three outcomes. That is, if data is missing for a specific outcome for a patient, that patient will not contribute to the corresponding analysis. For potential prognostic factors, a mixed approach will be used: if more than 90% of patients per centre have complete data for all factors for a given analysis, then complete case analysis will be used as the primary analysis for that centre. If not, missing value imputation [35] will be used according to the framework set out below before any models are fitted, and complete case analysis will be performed as a robustness check. Missing data imputation has only been sparsely explored in the context of distributed learning and there is only limited precedence to guide best practise [36, 37]. Initially, we will implement the missing data imputation framework described below, but this may be adapted based on our ability to implement more robust techniques in a distributed setting.

Where data for potential prognostic factors is missing for a small number of patients in individual centres (> 10% but ≤ 50%), local data imputation techniques will be employed. Missing data will be imputed using only the local dataset, and prior to any distributed model optimisation. The k-nearest neighbour (KNN) algorithm [38, 39] will be used to carry out the imputation [40]. Using this algorithm, each missing value will be replaced by a value that is as close as possible to the true value, obtained from related cases in the whole dataset. This technique aims to preserve the original structure of the dataset and avoids distorting the distribution of the imputed data item. To implement KNN, an appropriate value of k will be first determined through exploration of the data in each centre, using the square root of the sample size as a starting point [38, 41]. All available essential data items, as well as outcome data [42], will be included in the imputation model.

Where data for potential prognostic factors is systematically missing in specific centres (> 50% data missing for any specific item), the general assumption will be that imputation based on the local centre data will be unreliable. In this case, consortium-wide regression will be implemented to impute the missing data items.

As an initial plan, a regression model will first be trained using data from all centres apart from the centre where the data item is missing. This model will then be run in the centre where the data is missing to impute the missing values. If two or more centres are missing the same data item, this approach will not be technically feasible due to limitations of the DL infrastructure that will be used. In this case, for continuous data items, the mean from each centre (apart from the centres with missing data) will be used to calculate the global “median of means” value for that data item. This value will be assigned to all patients in the centres where the data item is missing. For categorical data items, the frequency of each category across the global cohort for the data item that is missing will be calculated (excluding centres with the missing data item). Categories will then be assigned to each patient at random in centres where the data item is missing, ensuring the local frequency distribution is the same as the global frequency distribution.

In parallel to the main consortium analysis, an independent exploratory study will be carried out to evaluate the feasibility of various imputation techniques in the context of distributed learning. Once feasible and robust techniques have been identified, we will look at implementing these to improve on the missing data imputation framework described above.

### Sample size

The atomCAT1 proof-of-concept study [31] demonstrated the ability to combine data from three centres for 281 patients. A Cox regression model for overall survival was fitted to the global dataset, taking five baseline factors into account. Its performance was evaluated using Harrell’s concordance index (c-index). The internal-external validation approach returned a c-index of 0.70, which is considered as “good” model performance.

The “pmsampsize” [43] package in R was used to estimate the minimum sample size required for the atomCAT2 models. The parameters required to carry out this calculation include *R*^2^ (calculated from the c-index), number of candidate predictor parameters, shrinkage (level of reduction of the estimated predictor effect estimates to address overfitting), overall event rate in the population, mean follow-up time anticipated for individuals in the model development dataset and timepoint of interest for prediction.

Table 2 illustrates how many patients will be needed to fit a Cox proportional hazards model for overall survival in atomCAT2, aiming for similar performance to the model developed in the atomCAT1 study, with varying number of parameters. These estimates assume an event rate of 16% at 36 months, with a median follow-up of 46 months, a c-index of 0.70 (corresponding to *R*^2^_CSapp_ of 0.0676) and a shrinkage value of 0.90. Sample size estimates in this setting are very robust to variations in event rate, and are thus also valid for models for locoregional control and freedom from distant metastases (with an event rate of 25% and 15% at 5 years, respectively [7]).

**Table 2:** Estimated minimum sample size for a range of parameters, for the overall survival model (also valid for the locoregional control and freedom from distant metastasis models)

Parameters included in the model	Minimum sample size
5	641
6	769
7	897
8	1025
9	1153
10	1283
11	1409

The number of prognostic factors which will be included in the final models will be based on the total number of patients available across the consortium. The analysis plan will be finalised when the total number of available patients are confirmed. Currently, we aim to include data from at least 1000 patients, which would allow for eight parameters to be estimated per model. The number of prognostic factors included in the model could be the same or different to the number of parameters depending on the number of categories for the categorical factors and the parameterisation of the continuous factors.

### Statistical analysis

#### Descriptive data analysis

Summary statistics will be shared with the central study team in order to explore cohort differences prior to modelling. Categorical variables will be summarised as proportions to the total number of patients per centre, expressed as percentages. Summary statistics will be calculated for numerical variables (mean, standard deviation, range, variance).

Summary statistics for the global cohort will be reported. Categorical variables will be summarised as proportions to the total number of patients in the global cohort, expressed as percentages. For numerical variables, random effect meta-analysis will be used (using the “meta” package in R), with inverse variance weighting for pooling, reporting the overall mean and 95% confidence intervals (calculated from overall standard deviations). The range will be reported as the lowest and highest values across the global cohort, calculated from the range from each centre.

Estimated 3-year survival/freedom from recurrence rates will be calculated by each centre individually, using the Kaplan-Meier estimator (using the “survival” package in R). The median potential follow-up time will be calculated based on the inverse Kaplan-Meier estimator for each outcome for each centre separately.

#### Model specification

The prognostic factors that will be included in each of the three primary models are specified in Table 3. The factors are listed in the order that they will be prioritised for analysis based directly on the findings from the systematic review and expert opinion. The factors are ordered according to the total number of times found to be prognostic in multivariable analysis. Additional factors were added by senior clinical oncologists. Factors found to be prognostic in univariable analysis but not in multivariable analysis may be included in the secondary models. The number of factors included in each model will depend on the final sample size for each outcome, as detailed above. For each factor, the primary parameterisation used (e.g. categorisation for categorical variables) is listed, with alternatives to be explored in secondary analyses. The parameterisation of all variables for the primary and secondary models was determined after a detailed discussion with clinical oncologists and represents the relationship they expect to see from clinical experience, as well as the expected data distribution. See Additional file 2 for secondary model specification.

**Table 3:** Specification of the primary models for overall survival, locoregional control and freedom from distant metastasis

Prognostic factors to be included in the primary models			
Overall survival model		Locoregional control model	Freedom from distant metastasis model
1	N stage: N0 vs N+	Sex: female vs male	N stage: N0 vs N+
2	T stage: T1–2 vs T3–4	N stage: N0 vs N+	T stage: T1–2 vs T3–4
3	Sex: female vs Male	T stage: T1–2 vs T3–4	Sex: female vs male
4	Age: modelled as a continuous, linear factor	Age: modelled as a continuous, linear factor	Age: modelled as a continuous, linear factor
5	Primary tumour GTV (cm^3^): modelled as a continuous, log-transformed factor	Primary tumour GTV (cm^3^): modelled as a continuous, log-transformed factor	Primary tumour GTV (cm^3^): modelled as a continuous, log-transformed factor
6	Primary tumour dose (EQD2): modelled as a continuous, linear factor	Primary tumour dose (EQD2): modelled as a continuous, linear factor	Primary tumour dose (EQD2): modelled as a continuous, linear factor
7	Histology: SCC vs basaloid SCC	Histology: SCC vs basaloid SCC	Histology: SCC vs basaloid SCC
8	Chemotherapy regimen: [no chemotherapy] vs [mitomycin C-based regimen] vs [cisplatin-based regimen]	Chemotherapy regimen: [no chemotherapy] vs [mitomycin C-based regimen] vs [icsplatin-based regimen];	Chemotherapy regimen: [no chemotherapy] vs [mitomycin C-based regimen] vs [cisplatin-based regimen];
9	RT technique: [3D-CRT] vs [IMRT] vs [VMAT]	RT technique: 3D-CRT vs IMRT vs VMAT	RT technique: 3D-CRT vs IMRT vs VMAT

*N stage* nodal stage, *T stage* tumour stage, *GTV* Gross tumour volume, *EQD2* Equivalent dose in 2 Gy fractions (α/β = 10 Gy), *SCC* Squamous cell carcinoma, *3D-CRT* Three-dimensional conformal radiation therapy, *IMRT* Intensity-modulated radiation therapy, *VMAT* Volumetric modulated arc therapy

#### Cox model development and reporting

The primary analysis will involve the development and internal validation (type 2b validation according to TRIPOD [44]) of Cox proportional hazards models using distributed learning [45] across all participating centres, separately for each outcome (overall survival, locoregional control, and freedom from distant metastasis). The primary model to be developed for each outcome is detailed above. Secondary analyses (Additional file 2) will be used to explore the robustness of the results to the choices made for the primary model. As an additional assessment of model robustness, another secondary analysis will be conducted. In this analysis, the specified models (Table 3) will be trained only on datasets comprising of more than 20 events, as a way of testing whether the number of events per centre affects the behaviour of the models.

The factor effects from each model will be reported in the form of hazard ratios (HRs) along with 95% confidence intervals (CIs). The ‘baseline’ outcome rate at specific timepoints of interest (e.g. 2 years, 3 years and 5 years) will be calculated. Combining the baseline outcome rate with the factor effect estimates (HRs) will allow for outcome prediction for a future patient, rendering the model useable for future prediction.

### Evaluation and visualisation of model performance

Model performance will be initially assessed using Harrell’s concordance index (c-index) [46] on a per-centre basis, with a weighted average c-index (and standard deviation) also reported. A more robust estimate for out-of-sample performance will be obtained using a closed-loop internal-external “leave-one-centre-out” cross-validation method [47], where the model will be optimised using data from all but one sites and then validated on the last site. This will be repeated to cover the possible combinations, resulting in different c-indices which provide an estimate of the over-optimism of the global model. The weighted average and interquartile range (IQR) of these c-index values will be reported. The factor effects from each of these validation models will be aggregated and the summary effects will be reported in the form of HR range for each factor across all models.

The calibration of the global model (performance check for the prediction aspect of the model) will be assessed by constructing calibration curves and quantifying the calibration slope [48, 49], on a local (per-centre) level. Calibration curves will use three groups per centre (low/medium/high risk, based on their predicted outcomes), and will compare average predicted outcome within each group with the observed outcome at 3 years, using the Kaplan-Meier estimator. This is the initial plan for evaluation of the model calibration, and the final plan may be altered depending on the size of each centre’s cohort, as well as the number of events per centre.

The model development and validation procedure and results will be reported in accordance with the TRIPOD statement and checklist [50]. This protocol has also been checked against the relevant parts of the TRIPOD checklist for prediction model development and validation (see Supplementary Material).

### Distributed learning infrastructure

The infrastructure that will be used for this study is very similar to the infrastructure implemented in atomCAT1 [31]. The Distributed Cox algorithm developed by Lu et al. [45] was adapted to the Vantage6 v2.1 infrastructure as R scripts (v.3.6.2). The source code will be made openly accessible on GitHub. Scripts for computing model coefficients and leave-one-centre-out model validation will be packaged as application “containers” (via Docker) and will be locally executed in each centre. All other scripts that will be used for the data analysis will be uploaded in a GitLab repository, which will be made public at the end of the project.

### Organisation and policies

The atomCAT2 study will be conducted as part of a wider atomCAT consortium. Details of consortium engagement and project management will be described in detail in a collaborative research agreement, which will be signed by all participating centres.

Medical Data Works BV (MDW, https://medicaldataworks.nl/) implements a privacy preserving distributed infrastructure that investigators in atomCAT2 will use. Therefore, an Infrastructure User Agreement will be signed as a contractual agreement between each centre and MDW. MDW will not be considered as a “processor” of clinical data according to the definition in the EU General Data Protection Regulation but is solely the provider of the information technology infrastructure and the central server. As the infrastructure provider, MDW will enforce the legal use of algorithms and data stations, and this agreement shall define the terms and conditions for the use of the infrastructure.

## Discussion

This paper describes the protocol and statistical analysis plan for the international multi-centre atomCAT2 study. The study will aim to develop and validate robust prognostic models for three clinically important outcomes in anal cancer after treatment with conformal radiotherapy. Key prognostic factors for each outcome will also be identified and validated.

Only patients treated with conformal radiotherapy techniques (e.g. 3D-conformal, intensity-modulated radiation therapy (IMRT) and volumetric modulated arc therapy (VMAT)), will be included in the cohort for analysis as these techniques have been proven to reduce the dose delivered to normal tissues, minimising toxicity and reducing overall treatment duration and the need for treatment breaks [51–54]. Therefore, by limiting our cohort to patients treated with conformal radiotherapy, we ensure that the prognostic models developed will be informative to current clinical practice. These models will include a range of established prognostic factors, identified through a comprehensive review of the literature and confirmed by three experts from three different centres. A range of additional less-established prognostic factors will also be tested in secondary models, to quantify their effect size and assess their eligibility as clinically relevant predictors of outcome.

Most of the literature which reports on outcomes and prognostic factors in anal cancer after conformal radiotherapy are retrospective studies which include small cohorts from a single centre. The results from the prognostic models developed in these studies may therefore suffer from small sample size bias and might not be generalisable [55] across centres and countries. To our knowledge, only three previous studies have analysed more than 200 patients with conformal radiotherapy [14, 31, 56], only one of which was multi-national and conducted multi-centre validation of outcome predictors. The cohorts that will be included in atomCAT2 will not only be significantly larger in size, but also more heterogeneous, since treatment dose and delivery schedules vary between radiotherapy centres, especially across different countries.

The analysis will be limited to retrospective data that is readily available in clinical and radiotherapy planning databases in a large number of centres. Therefore, some factors that could potentially be prognostic, such as HPV status and baseline performance status, may not be included in the primary models as they are not routinely collected in all centres. Since atomCAT2 is a non-prospective multi-centre analysis, it is expected that some data will vary between centres, including tumour staging, GTV definitions and outcome definitions. Steps have been taken to take the variation into consideration and minimise it as much as possible, including providing pre-specified definitions for all three outcomes and asking centres to indicate the staging version and GTV definition used. Despite this, some variation is unavoidable, which may affect the results. Additionally, it is expected that some essential data will be missing in a number of centres.

The methods for handling missing data have been specified in the protocol, however, these are substantially limited to what can currently be implemented in the DL setting without having to share individual-level patient data between centres. The field of missing data imputation in the context of DL is still in its infancy and does not currently have established standards. So far, only few studies have been conducted with the aim of developing or evaluating imputation techniques that can be implemented in a DL setting [36, 37]. Our initial imputation plan for data missing for a small number of patients in individual centres proposes the implementation of the KNN algorithm, which is a single imputation approach. In this case, one unique value will be imputed for each patient with missing data, resulting in a single complete dataset [57, 58]. This will likely produce relatively unbiased estimates, especially if only a small proportion of the data is missing [57, 59]. However, it is worth noting that these approaches fail to take into account the uncertainty of the missing values [60], which often results in underestimation of the variability and standard errors that are too small [61]. If data for a single data item is missing in the majority of patients in an individual centre, we also propose single imputation (using the data from the remaining centres), but assigning the same value to all missing data from the centre in question (also referred to as single value imputation). We recognise that this approach may introduce significant bias, leading to a change in the distribution shape and a significant decrease in standard deviation of the data item being imputed [62]. Using more advanced approaches to impute missing data, such as multiple imputation by chained equations (MICE) [63], would be ideal but cannot be applied through the DL infrastructure at this point. Further methodological research is needed to incorporate robust data imputation techniques to a privacy-preserving setting in order to tackle the problem of missing data, which is particularly common in medical datasets.

Future research beyond atomCAT2 will include incorporation of imaging and radiomics data to the models to increase their complexity and the potential insights gained. A number of studies have reported various imaging-related prognostic factors in anal cancer [64–66], which might prove to be clinically relevant. Moreover, strong efforts from the research community are being put into increasing the utility of DL in medical research by adapting different statistical methods and models to the existing infrastructure. In the future, it may be possible to develop competing risk models [67] in a distributed fashion, allowing multiple outcomes to be analysed in combination. Additionally, other algorithms such as random survival forests [68], may be implemented in DL to carry out the analysis instead of Cox regression. Random survival forests allow for a larger number of factors to be considered and factor selection is embedded within the methodology, which may in turn improve learning performance. This will be particularly useful in cases where many factors need to be considered. Alternative approaches to DL could also be considered for prognostic model development without having to share individual-level patient data between centres. For example, a multivariate meta-analysis approach [69–71] could be adopted, where summary statistics and regression coefficients from different prognostic models can be combined into a new prediction model. However, there are various issues with this approach, which may have a negative impact on the performance of the resulting prediction model, such as inconsistent covariate adjustment across models and high levels of model heterogeneity [72]. One significant advantage of the distributed learning approach over a meta-analysis approach is that a distributed Cox regression model generates the same model outputs as a centralised Cox regression model trained with the same data [45]. It has also been proven that distributed and centralised Cox regression models are equivalent from a mathematical perspective. This might not be true in all cases where meta-analysis approaches to prognostic model development are employed.

In conclusion, the atomCAT2 models will be developed using one of the largest cohorts of patients with anal cancer treated with conformal radiotherapy techniques ever analysed and will be validated across centres and countries. The models will allow for the prediction of outcomes in individual patients, which will inform current clinical practice and may subsequently aid with the personalisation of anal cancer treatment. The results of the atomCAT2 study may guide patient risk stratification, which may in turn facilitate the design of future prospective clinical trials in anal cancer.

## Supplementary Information

**Additional file 1: Appendix 1. Data dictionary**

**Additional file 2: Appendix 2. Specification of secondary models**

## Data Availability

Due to the nature of this research, the datasets that will be analysed during the study will not be made available, as no patient consent or ethics approval could be acquired for individual-level patient data sharing. All results generated during the study will be made available as part of the final publication.

## Abbreviations

3D-CRT: Three-dimensional conformal radiation therapy
ASCC: Anal squamous cell carcinoma
atomCAT: Anal cancer Treatment Outcome Modelling with Computer Aided Theragnostics
CI: Confidence interval
CT: Computed tomography
DL: Distributed learning
EQ D2: Equivalent dose in 2 Gy fractions (α/β = 10 Gy)
GTV: Gross tumour volume
HR: Hazard ratio
IG: Information governance
IMRT: Intensity-modulated radiation therapy
IQR: Interquartile range
IRB: Institutional review board
KNN: k-Nearest neighbour algorithm
MDW: Medical Data Works
MICE: Multiple imputation by chained equations
MLCs: Multi-leaf collimators
VMAT: Volumetric modulated arc therapy
