# Supplementary material for: Development and validation of prognostic models for anal cancer outcomes using distributed learning: protocol for the international multi-centre atomCAT2 study
Source: Diagn Progn Res. 2022 Aug 4;6:14. doi: 10.1186/s41512-022-00128-8 (PMC9351222; doi:10.1186/s41512-022-00128-8)
Supplement: Supplementary file 1 — Additional file 1: Appendix 1. Data dictionary [file 41512_2022_128_MOESM1_ESM.docx]

### Additional File 1. Data dictionary

Essential data items are denoted in **bold**. All other data items are optional.

All missing values will be coded as **NA**.

#### Baseline characteristics

- **Biological sex** [*sex*]: Binary variable
  - 0–Male
  - 1–Female
- **Age at the start of radiotherapy** (years) [*age*]: Continuous numerical variable
- **TNM staging**: Categorical variables
  - **T stage** [*t_stage*]
    - 1: T1
    - 2: T2
    - 3: T3
    - 4: T4
  - **N stage** [*n_stage*]
    - for TNM version 7: 0: N0; 1: N1; 2: N2; 3: N3
    - for TNM version 8: 0: N0; 1: N1a; 2: N1b; 3: N1c
  - **M stage** [*m_stage*]
    - 0: M0
    - 1: M1
- **TNM staging version** [*tnm_version*]: Discrete numerical variable
- **Primary tumour GTV** (cm^3^) [*pr_tumour_gtv*]: Continuous numerical variable
- **Histology** [*histology*]: Binary variable
  - 0–SCC
  - 1–Basaloid SCC
- HPV status [*hpv_status*]: Binary variable
  - 0–Negative
  - 1–Positive
- Performance status [*perf_status*]: Categorical variable
  - 0–Fully active, able to carry on all pre-disease performance without restriction.
  - 1–Restricted in physically strenuous activity but ambulatory and able to carry out work of a light or sedentary nature, e.g., light housework, office work.
  - 2–Ambulatory and capable of all self-care but unable to carry out any work activities. Up and about more than 50% of waking hours.
  - 3–Capable of only limited self-care, confined to bed or chair more than 50% of waking hour.
  - 4–Completely disabled. Cannot carry on any self-care. Totally confined to bed or chair.
- Metastasis site at diagnosis [*met_site_diag*]: Categorical variable
  - 0–No distant metastasis
  - 1–Lymph nodes outside pelvis
  - 2–Viscera or bones
  - 3–Multiple sites
- GTV delineation definition [*gtv_definition*]: Categorical variable
  - - This variable does not need to be assessed for each patient individually, only on a per-centre level.
  - 1–Primary tumour only
  - 2–Primary tumour and anal canal in areas of tumour involvement
  - 3–Primary tumour and entire anal canal
- Differentiation grade [*diff_grade*]: Categorical variable
  - 0–Well differentiated
  - 1–Moderately differentiated
  - 2–Poorly differentiated

#### Treatment-related factors

- **Radiotherapy technique** [*rt_technique*]**:** Categorical variable
  - 1–3D-CRT
  - 2–IMRT
  - 3–VMAT
- **Total prescribed dose** (in EQD2_α/β=10Gy_): Continuous numerical variable
  - **To primary tumour** [*prescr_dose_prtumour*]
  - **To involved lymph nodes** [*prescr_dose_invnodes1, prescr_dose_invnodes2*]
  - **To elective nodes** [*prescr_dose_elenodes1, prescr_dose_elenodes2*]
- **Concurrent chemotherapy?** [*conc_chemo*]: Binary variable
  - 0–No
  - 1–Yes
- **Concurrent chemotherapy–number of cycles** [*conc_chemo_cycles*]: Discrete numerical variable
- **Concurrent chemotherapy–drugs used** [*conc_chemo_drugs*]: Categorical variable
  - 0–No chemotherapy
  - 1–Mitomycin C and 5-Fluorouracil
  - 2–Mitomycin C and Capecitabine
  - 3–Cisplatin and 5-Fluorouracil
  - 4–Cisplatin and Capecitabine
  - 5–Other
- Total number of prescribed treatment fractions [*prescr_fractions*]: Discrete numerical variable
- Total number of delivered treatment fractions [*deliv_fractions*]: Discrete numerical variable
- Overall treatment time (days) [*overall_treatment_time*]: Discrete numerical variable
- Completed radiotherapy treatment? [*compl_treatement*]: Binary variable
  - 0–No
  - 1–Yes
- Treatment breaks? [*treatment_breaks*]: Binary variable
  - - Defined as any extension to the treatment time of more than 2 days over the planned overall treatment time (as defined by RCR [1])–extensions due to planned breaks such as holidays should not be included.
    - Estimated from the total number of delivered fractions - gives expected treatment time-compared to overall treatment time
    - We do not need chart checks for breaks for all patients, but just an estimate of whether treatment time is extended compared to expected
  - 0–No
  - 1–Yes
- Simultaneous or sequential boost? [*boost*]: Categorical variable
  - 0–No boost
  - 1–Simultaneous boost
  - 2–Sequential boost
- Total delivered dose (in EQD2_α/β = 10 Gy_): Continuous numerical variable
  - To primary tumour [*deliv_dose_prtumour*]
  - To involved lymph nodes [*deliv_dose_invnodes1, deliv_dose_invnodes2*]
  - To elective nodes [*deliv_dose_elenodes1, deliv_dose_elenodes2*]

#### Outcomes

- **Overall survival status** [*os_status*]: Binary variable
  - 0–Alive
  - 1–Dead
- **Overall survival—follow-up time (days)** [*os_fup*]: Discrete numerical variable
  - Calculated in number of days from the first fraction of radiotherapy to either event or censoring, whichever happens first.
- **Locoregional failure** [*lrf_status*]: Binary variable
  - 0–No
  - 1–Yes
- **Site of locoregional failure** [*lrf_site*]: Categorical variable
  - 0–No locoregional failure
  - 1–Primary tumour
  - 2–Pelvic lymph nodes/lymph nodes in the primary treatment volume
  - 3–Primary tumour and lymph nodes simultaneous
  - 4–Other
- **Locoregional failure—follow-up time (days)** [*lrf_fup*]: Discrete numerical variable
  - Calculated in number of days from the first fraction of radiotherapy to either event or censoring, whichever happens first.
- **Distant metastasis** [*dm_status*]: Binary variable
  - 0–No
  - 1–Yes
- Site of distant metastasis [*dm_site*]: Categorical variable
  - 0–No distant metastasis
  - 1–Lymph nodes outside pelvis
  - 2–Viscera or bones
  - 3–Multiple sites
- **Distant metastasis—follow-up time (days)** [*dm_fup*]: Discrete numerical variable
  - Calculated in number of days from the first fraction of radiotherapy to either event or censoring, whichever happens first.

#### Availability of imaging and treatment plans

In future analysis, baseline imaging biomarkers will be explored and incorporated to the models. The availability of the following factors will be assessed in each centre:

- Pre-treatment FDG PET-CT scan available: Binary variable
  - 0–No
  - 1–Yes
- Pre-treatment MRI scan available: Binary variable
  - 0–No
  - 1–Yes
- Treatment planning CT scan available: Binary variable
  - 0–No
  - 1–Yes
- Full 3D dose distributions for all treatment phases available: Binary variable
  - 0–No
  - 1–Yes
- Radiotherapy structure set data available: Binary variable
  - 0–No
  - 1–Yes
- Dose-volume histogram (DVH) data available: Binary variable
  - 0–No
  - 1–Yes

#### References

[1] London: Royal College of Radiologists. The timely delivery of radical radiotherapy: Standards and guidelines for the management of unscheduled treatment interruptions. 2008. <https://www.rcr.ac.uk/publication/timely-delivery-radical-radiotherapy-guidelines-management-unscheduled-treatment>. Accessed 25 Feb 2022.
