# Supplementary material for: Development and validation of prognostic models for anal cancer outcomes using distributed learning: protocol for the international multi-centre atomCAT2 study
Source: Diagn Progn Res. 2022 Aug 4;6:14. doi: 10.1186/s41512-022-00128-8 (PMC9351222; doi:10.1186/s41512-022-00128-8)
Supplement: Supplementary file 2 — Additional file 2: Appendix 2. Specification of secondary models [file 41512_2022_128_MOESM2_ESM.docx]

### Additional File 2. Specification of secondary models

#### Overall survival secondary models

A range of secondary models for overall survival will be fit, in order to test the robustness of the primary model, as well as explore the impact of having a different set of factors or different factor parameterisation on the model fit. The following changes will be made to the primary model in separate secondary models:

1. TNM staging: will replace T stage and N stage. Categories: Low risk (T1-3N0) vs High risk (T4N(any) or T(any)N+).
2. Age: Modelled as a categorical factor instead of continuous. Categories: [18–39] vs [40–59] vs [60–79] vs [80–99].
3. Age: Modelled as a continuous, non-linear factor. Multiple transformations will be tested prior to the analysis and the most appropriate transformation will be applied.
4. Primary tumour GTV (cm^3^): Modelled as a categorical factor instead of continuous. Categories: [0–49.99] vs [50–99.99] vs [100–149.99] vs [150–199.99] vs [200+].
5. Chemotherapy regimen: will be included as a factor with 5 categories. Categories: [No chemotherapy] vs [Mitomycin C and 5-Fluorouracil] vs [Mitomycin C and Capecitabine] vs [Cisplatin and 5-Fluorouracil] vs [Cisplatin and Capecitabine] vs [Other].
6. Incomplete/Interrupted treatment will be included as a binary factor: No vs Yes
7. Performance status will be included as a categorical factor: 0 vs 1 vs 2 vs 3 vs 4 (see Appendix 1 for categorisation).

Note: Items 6 and 7 were found to be prognostic for overall survival in univariable analysis in the systematic review, but not in multivariable analysis. These are *optional* data items in atomCAT2. We will assess the amount of data available and if possible, secondary models which include these factors will be fit.

#### Locoregional control secondary models

A range of secondary models for locoregional control will be fit. The following changes to the primary model will be made in separate secondary models:

1. Performance status will be included as a categorical factor: 0 vs 1 vs 2 vs 3 vs 4 (see Appendix 1 for categorisation).
2. TNM staging: will replace T stage and N stage. Categories: Low risk (T1-3N0) vs High risk (T4N(any) or T(any)N+).
3. Overall treatment time: will be included as a continuous, linear factor.
4. Age: Modelled as a categorical factor instead of continuous. Categories: [18–39] vs [40–59] vs [60–79] vs [80–99].
5. Age: Modelled as a continuous, non-linear factor. Multiple transformations will be tested prior to the analysis and the most appropriate transformation will be applied.
6. Primary tumour GTV (cm^3^): Modelled as a categorical factor instead of continuous. Categories: [0–49.99] vs [50–99.99] vs [100–149.99] vs [150–199.99] vs [200+].
7. Incomplete/Interrupted treatment will be included as a binary factor: No vs Yes.

Note: Items 1 and 7 were found to be prognostic for locoregional control in univariable analysis, but not in multivariable analysis. These are *optional* data items in atomCAT2. We will assess the amount of data available and if possible, secondary models which include these factors will be fit.

#### Freedom from distant metastasis secondary models

A range of secondary models for freedom from distant metastasis will be fit. The following changes to the primary model will be made in separate secondary models:

1. TNM staging: will replace T stage and N stage. Categories: Low risk (T1-3N0) vs High risk (T4N(any) or T(any)N+).
2. Age: Modelled as a categorical factor instead of continuous. Categories: [18–39] vs [40–59] vs [60–79] vs [80–99].
3. Age: Modelled as a continuous, non-linear factor. Multiple transformations will be tested, and the most appropriate transformation will be applied.
4. Primary tumour GTV (cm^3^): Modelled as a categorical factor instead of continuous. Categories: [0–49.99] vs [50–99.99] vs [100–149.99] vs [150–199.99] vs [200+].
5. Chemotherapy regimen: will be included as a factor with 5 categories. Categories: [No chemotherapy] vs [Mitomycin C and 5-Fluorouracil] vs [Mitomycin C and Capecitabine] vs [Cisplatin and 5-Fluorouracil] vs [Cisplatin and Capecitabine] vs [Other].
